# Mediterranean Diet Improves Plasma Biomarkers Related to Oxidative Stress and Inflammatory Process in Patients with Non-Alcoholic Fatty Liver Disease

**DOI:** 10.3390/antiox12040833

**Published:** 2023-03-29

**Authors:** Maria Magdalena Quetglas-Llabrés, Margalida Monserrat-Mesquida, Cristina Bouzas, Isabel Llompart, David Mateos, Miguel Casares, Lucía Ugarriza, J. Alfredo Martínez, Josep A. Tur, Antoni Sureda

**Affiliations:** 1 Research Group in Community Nutrition and Oxidative Stress, University of the Balearic Islands-IUNICS, 07122 Palma de Mallorca, Spain; m.quetglas@uib.es (M.M.Q.-L.); margalida.monserrat@uib.es (M.M.-M.); cristina.bouzas@uib.es (C.B.); isabel.llompart@ssib.es (I.L.); davidfrom13@gmail.com (D.M.); luciaugarriza@gmail.com (L.U.); antoni.sureda@uib.es (A.S.); 2 Health Research Institute of Balearic Islands (IdISBa), 07120 Palma de Mallorca, Spain; 3 CIBER Fisiopatología de la Obesidad y Nutrición (CIBEROBN), Instituto de Salud Carlos III (ISCIII), 28029 Madrid, Spain; jalfredo.martinez@imdea.org; 4 Radiodiagnosis Service, Red Asistencial Juaneda, 07011 Palma de Mallorca, Spain; casaresmiguel@gmail.com; 5 Clinical Analysis Service, University Hospital Son Espases, 07198 Palma de Mallorca, Spain; 6 Cardiometabolics Precision Nutrition Program, IMDEA Food, CEI UAM-CSIC, 28049 Madrid, Spain; 7 Department of Nutrition, Food Sciences and Physiology, University of Navarra, 31008 Pamplona, Spain

**Keywords:** non-alcoholic fatty liver disease, Mediterranean diet, metabolic syndrome, oxidative stress, inflammation

## Abstract

Non-alcoholic fatty liver disease (NAFLD) shows liver fat depots without alcohol consumption. NAFLD does not have specific drug therapies, with a healthy lifestyle and weight loss being the main approaches to prevent and treat NAFLD. The aim was to assess the antioxidant and pro-inflammatory state in patients with NAFLD after 12-month-lifestyle intervention depending on the change in adherence to a Mediterranean diet (AMD). Antioxidant and inflammatory biomarkers were measured in 67 adults (aged 40–60 years old) diagnosed with NAFLD. Anthropometric parameters and dietary intake were measured by a validated semi-quantitative 143-item food frequency questionnaire. The nutritional intervention improved anthropometric and biochemical parameters after a 12-month follow-up. However, decreases in alanine aminotransferase (ALT) and C reactive protein (CRP) were higher in participants with high AMD, which also showed higher improvement in physical fitness (Chester step test) and intrahepatic fat contents. The intervention reduced plasma levels of malondialdehyde, myeloperoxidase, zonulin, and omentin, and increased resolvin D1 (RvD1), whereas the decrease in leptin, ectodysplasin-A (EDA), cytokeratin-18 (CK-18), interleukin-1ra (IL-1ra) and endotoxin was only significant in participants with higher AMD. The current study showed that a one-year nutritional intervention improved main NAFLD features such as body mass index, IFC, liver enzymes, and prooxidant and proinflammatory status. There was also a decrease in the concentration of plasmatic endotoxin, suggesting an improvement in intestinal permeability. These health benefits were more evident in participants that improved AMD to a greater extent. The trial was registered at ClinicalTrials.gov with registry number NCT04442620.

## 1. Introduction

Non-alcoholic fatty liver disease (NAFLD) is associated with metabolic syndrome and it is characterized by increased intracellular lipid droplets exceeding 5% in absence of significant alcohol consumption [1]. NAFLD has been linked to several metabolic risk factors such as type 2 diabetes (47.3–63.7%) and obesity (80%) [2]. Nowadays, this disease is the most common cause of chronic liver disease, with a global prevalence of 25.2% [3]. Normally, this liver disease tends to remain stable or progress slowly, although it has been related to deaths from cardiovascular diseases and extrahepatic malignancies [4]. However, if the pathology is not reversed, it can evolve into more severe stages of the disease. In fact, about 10% of NAFLD patients develop advanced fibrosis, cirrhotic complications, and hepatocellular carcinoma within 10–20 years of disease diagnosis [4,5]. Overnutrition, in addition to promoting the expansion of adipose deposits, can favor an accumulation of ectopic fat and the infiltration of macrophages in the visceral adipose tissue compartment inducing a proinflammatory state and insulin resistance. This resistance has been associated with an inappropriate lipolysis and an imbalance in lipid metabolism leading to the formation of lipotoxic lipids and, consequently, to oxidative stress and endoplasmic reticulum stress [6]. 

One of the main problems in relation to NAFLD is its underdiagnosis, especially in the initial stages of the disease, since an effective diagnosis requires expensive or invasive methodologies. In this sense, the most reliable diagnosis of NAFLD is liver biopsy; however, as it is an invasive technique and a long-term disease, other methods such as ultrasound or magnetic resonance imaging (MRI) are usually used. Moreover, promising new blood biomarkers are being sought for adoption as a general diagnostic method [7].

To date, NAFLD is one of the chronic liver diseases that, despite being in advanced stages of development, do not yet have specific pharmacological therapies due to the complexity of its pathophysiology. However, it is well known that a healthy lifestyle and weight loss, are crucial for the prevention and treatment of NAFLD [8]. Several studies propose the Mediterranean diet (MedDiet) as a feasible and effective dietary pattern to prevent and/or reverse NAFLD due to its balanced composition of macronutrients [9,10]. In fact, the MedDiet pattern is directly recommended by the EASL-EASD-EASO Clinical as Choice for the Treatment of NAFLD [11]. MedDiet is based on a high intake of vegetables, fruits, legumes, whole grains, nuts, seeds, and extra virgin olive oil and a moderate consumption of red wine, and red meat. Due to the fact that MedDiet is rich in bioactive compounds such as phenolic compounds, with antioxidant, anti-inflammatory and antidiabetic effects, among others, several studies have shown how its adherence is related to an improvement in the inflammatory and oxidant state [12,13]. 

Due to the increasing prevalence of NAFLD and the lack of a specific treatment, this study aimed at the assessment of antioxidant and pro-inflammatory states using plasma and serum biomarkers in patients with NAFLD after 12 months of lifestyle intervention according to the adherence to a Mediterranean diet (AMD).

## 2. Methods 

### 2.1. Study Design and Participants

**Figure 1:**
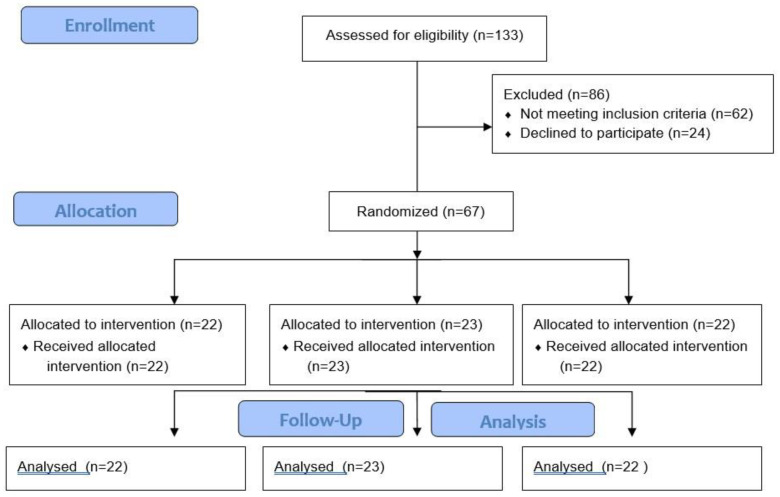
Flow diagram of the study.

A total of 67 participants ranging in age from 40–60 years with NAFLD diagnosed by Magnetic Resonance Imaging (MRI) were recruited in Mallorca Island, Spain Figure 1). To participate in the study, the inclusion criteria were the following: (1) body mass index (BMI) of 27–30 kg/m^2^ or an increased waist circumference of ≥94 cm in men and ≥80 cm in women; (2) triglycerides levels ≥150 mg/dL; (3) high-density lipoprotein cholesterol (HDL-c) <40 mg/dL in men and <50 mg/dL in women; (4) blood pressure ≥130/85 mmHg; (5) fasting serum glucose levels ≥100 mg/dL. The following exclusion criteria were applied: liver diseases (other than NAFLD); alcohol (>21 units a week for men and >14 for women) and drug abuse; nonmedicated depression or anxiety; previous cardiovascular disease; weight loss medications in the past 6 months; concomitant therapy with steroids; viral, autoimmune and genetic causes of liver disease; primary endocrinological diseases (other than hypothyroidism); pregnancy; previous bariatric surgery; active cancer or a history of malignancy in the previous 5 years. 

After inclusion, participants were randomly assigned to one of the following groups:

Conventional diet group (CD): participants followed the recommendations of the American Association for the Study of Liver Disease (AASLD) [14]. General guidelines from the US Department of Health and Human Services and the US Department of Agriculture (20–35% fat, 10–35% protein, 45–65% carbohydrates) indicate that from energy restrictions, a loss of at least 3–5% of body weight can be obtained, which can improve steatosis by 7–10%, thus improving the histopathological characteristics of NASH [15].

Mediterranean diet high meal frequency group (MD-HMF): participants followed a Mediterranean diet that had previously been shown to decrease fat mass and total weight and improve oxidative status in subjects with metabolic syndrome [16,17]. This diet is characterized by a macronutrient distribution of 40 to 45% carbohydrates (50 to 70% of carbohydrates should be low-glycaemic and high-fiber), 30 to 35% fat, and 25% protein. In addition, the daily caloric intake had to be distributed in a total of 7 meals, with the morning meals having the most calories.

Mediterranean diet physical activity group (MD-PA): participants followed an energy-restricted Mediterranean diet with a meal frequency of four to five meals per day. This diet is characterized by a macronutrient distribution of 40–45% carbohydrates (primarily low glycaemic index), 35–40% fat (8–10% saturated fatty acids, >20% monounsaturated fatty acids, >10% polyunsaturated fatty acids and <300 mg/day cholesterol) and about 20% protein. Also, sodium chloride intake should not reach 6 g/day (2.4 g sodium) and dietary fiber should not be less than 30–35 g/day [18].

The adherence of each of the participants to the MedDiet pattern was assessed using a validated questionnaire, as previously described [19]. The patients have been classified into two groups based on the degree of improvement in adherence to the MedDiet between the start of the study and 12 months after the intervention. Thus, the two groups would be made up of (1) those patients who have had a greater improvement in the degree of adherence and (2) made up of patients with a worse improvement at 12 months, according to the score. This form of grouping is because no differences were found between the different intervention groups since in all of them the different parameters of the participants were improved. For this reason, the grouping criteria were based on the degree of adherence to the MedDiet but considering the intervention group, for which the type of diet, frequency of feeding, and physical activity as co-variables in the statistical analysis.

The study protocols followed the Declaration of Helsinki ethical standards, and all the procedures were approved by the Ethics Committee of the Balearic Islands (CEIC- IB2251/14PI). All participants were informed of the purpose and the implications of the study, and informed consent was obtained from all subjects. This study has been registered in Clinicals Trials.gov ref. NCT04442620 [20].

### 2.2. Anthropometric Measurements

Professional dieticians carried out anthropometric measurements after identical and rigorous training to avoid interobserver bias. Body weight was determined without shoes using a Segmental Body Composition Analyzer (Tanita BC-418, Tanita, Tokyo, Japan) and 0.6 kg was subtracted for light clothing. Height was measured by keeping the patient’s head in the Frankfort Horizontal Plane position with a mobile anthropometer (Seca 214, SECA Deutschland, Hamburg, Germany). With both measurements, it was calculated BMI (kg/m^2^). Blood pressure was measured with a validated semi-automatic oscillometer (Omron HEM, 750CP, Hoofddrop, The Netherlands) in triplicate while the patient was sitting. The maximal oxygen uptake (VO_2_ max) was measured with the Chester step test (CST) [21]. Intrahepatic fat content (IFC) was performed with a 1.5-T MRI (Signa Explorer 1.5T, General Electric Healthcare, Chicago, IL, USA) by using a 12-channel phased-array coil [22]. 

### 2.3. Blood Collection and Analysis

After 12 h of overnight fasting, blood samples were collected from the antecubital vein in suitable vacutainers with ethylenediaminetetraacetic acid (EDTA) as an anticoagulant to obtain plasma and other vacutainers without anticoagulant to obtain serum. To obtain plasma samples, fresh blood was centrifugated at 1700× *g* 15 min at 4 °C. General blood biochemical analyses on serum in a fasting situation were performed by standard enzymatic methods in the clinical laboratory of Hospital de Son Espases (Palma de Mallorca). The parameters determined in serum were glucose, HbA1c, triglycerides, HDL-c, low-density lipoprotein cholesterol (LDL-c), total cholesterol, aspartate aminotransferase (AST), alanine aminotransferase (ALT), gamma-glutamyl transferase (GGT) and c-reactive protein (CRP) were determined using standardized clinical procedures. In a Technicon H2 VCS system automatic flow cytometer analyser (Bayer, Leverkusen, Germany), haematological parameters and complete blood count were determined.

### 2.4. Enzymatic Determinations

The activities of catalase (CAT) and superoxide dismutase (SOD) were determined in plasma and measured at 37 °C with a Shimadzu UV-2100 spectrophotometer (Shimadzu Corporation, Kyoto, Japan). CAT activity was measured using Aebi’s spectrophotometric method based on the decomposition of H_2_O_2_ at 240 nm [23] whereas SOD activity was determined by an adaptation of McCord and Fridovich’s method at 550 nm [24].

### 2.5. Malondialdehyde Assay

Malondialdehyde (MDA) was measured using the specific colorimetric assay kit (Merck KGaA^®^, Madrid, Spain) and the absorbance was measured at 586 nm following the manufacturer’s instructions. 

### 2.6. Phenolic Compounds Determination

Plasma samples were deproteinized with cold acetone (1:1.2) to determine the content of total phenolic compounds using the method of Folin–Ciocalteau [25] and using L-tyrosine as standard. The results are expressed as mmols of L-tyrosine equivalents/L.

### 2.7. Immunoassay Kits

Xanthine oxidase (XOD), myeloperoxidase (MPO), and zonulin plasma levels were measured using ELISA kits (Cusabio^®^ Technology Llc, Houston, TX, USA). Resolvin D1 (RvD1) levels were measured in plasma using an ELISA kit (Cayman Chemical^®^, Ann Arbor, MI, USA). Cytokeratin 18 (CK-18) levels were measured also in plasma using an M30 Apoptoense^®^ ELISA kit (VLVbio AB, Nacka, Sweden) in which the units measured are defined against a native antigen, and it is calibrated against a recombinant protein standard that 1 U/L = 1.24 pM. Ectodysplasin-A (EDA) plasma levels were determined using an ELISA kit (Assay Genie, Dublin, Ireland). Chemerin and omentin levels were measured in plasma and in serum respectively using ELISA kits (Abcam^®^, The Netherlands, Amsterdam). IL-1β, IL-1ra, IL-6, MCP-1, TNFα and leptin levels were determined in plasma using Human Custom ProcartaPlex^TM^ (Invitrogen by Thermo Fisher Scientific, Bender MedSystems GmbH, Vienna, Austria). The concentrations of endotoxin were measured in plasma by a commercially available kit Εndotoxin (Abbexa Ltd., Cambridge Science Park, Cambridge, UK). All ELISA kits were carried out following the supplier’s guidelines for use.

### 2.8. Statistical Analysis

Analyses were performed with the Statistical Package for Social Sciences (SPSS v.27, IBM Software Group, Chicago, IL, USA). The sample size for the original trial was estimated using weight loss as main outcome assuming a two-group *t*-test (two-sided) of difference between CD and the other groups. Results are expressed as the mean ± standard deviation (SD), and *p* < 0.05 was considered statistically significant. A Kolmogorov–Smirnov test was previously applied to assess normality. Two types of statistics were performed to check the significance of the resulting data. First, a two-way analysis of covariance (ANCOVA) was performed after adjustments by the intervention (diet and physical activity) or a Kruskal–Wallis test according to the case. Bonferroni post hoc analysis was conducted. Second, *t*-test for unpaired data was performed on the differential data (12-month values minus reference values) or with U Mann–Whitney test according to the case.

## 3. Results

**Figure 2:**
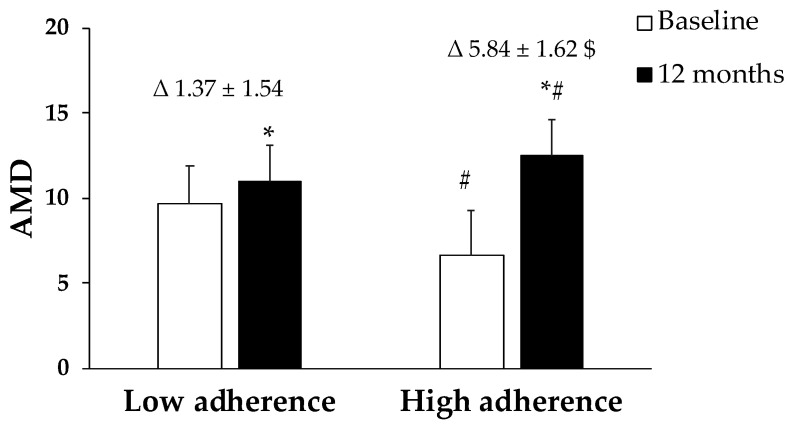
Adherence to Mediterranean Dietary (AMD) expressed as points obtained in the questionnaire. Results are presented as mean ± SD. Two-way analysis of co-variance (ANCOVA) after adjustments by the intervention (diet and physical activity). AMD × T interaction between adherence to the Mediterranean Diet and time. * Difference in means between participants in time (baseline and 12 months). ^#^ Difference in means between groups (low adherence and high adherence). *t*-test for unpaired data in the differential values. ^$^ Difference in means according to the differential (12-month values minus baseline values). Data points are significant when *p* < 0.05.

Figure 2 shows the change in adherence to MedDiet after 12 months of lifestyle intervention. The first group, named as low adherence, showed a variation in the AMD from 9.63 ± 2.26 to 11.0 ± 2.56, while the second group, high adherence, changed from 6.66 ± 2.11 to 12.5 ± 2.08.

The anthropometric and clinical characteristics of participants with NAFLD stratified by the AMD after a nutritional intervention for 12 months are shown in Table 1. The group that after the 12-month intervention acquired a greater AMD obtained a greater weight loss and a reduction in BMI in relation to the other group. Significant differences were evidenced in a decrease in AST, ALT, and CRP levels when comparing the evolution after 12 months of acquisition of greater AMD. Also, it was evidenced that systolic blood pressure was reduced in both groups after a nutritional intervention of 12 months regardless of the level of adherence to AMD. Triglycerides and cholesterol total levels were significantly reduced in the group with high AMD, even though they started with higher levels than the other group. Other parameters analyzed did not show significant differences between the time and the two groups of AMD.

**Figure 3:**
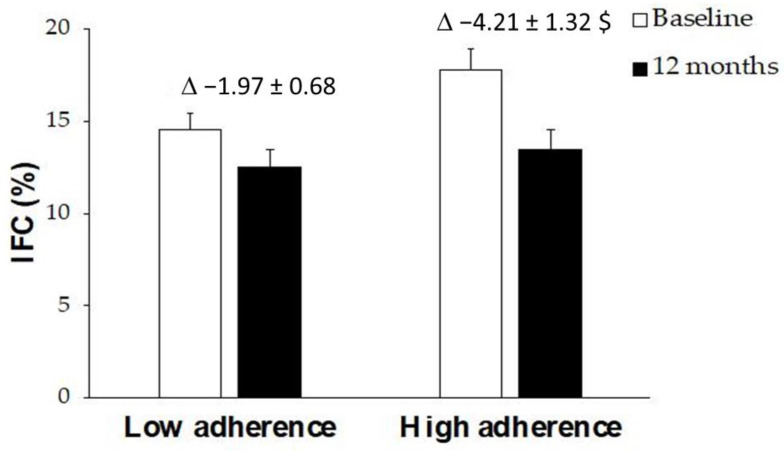
Percentage of IFC in patients with NAFLD at ‘Baseline’ and ‘12 months’ stratified by AMD. Results are presented as mean ± SD. Two-way analysis of co-variance (ANCOVA) after adjustments by intervention (diet and physical activity). AMD × T interaction between adherence to the Mediterranean Diet and time. *t*-test for unpaired data in the differential values. ^$^ Difference in means according to the differential (12-month values minus baseline values). Data points are significant when *p* < 0.05.

**Figure 4:**
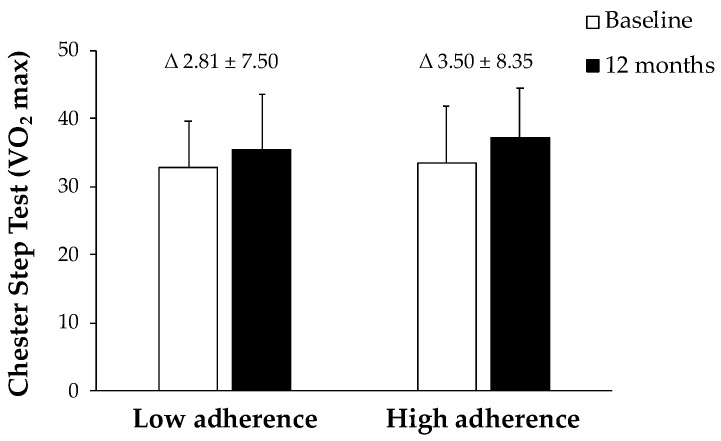
VO_2_ maximum values in patients with NAFLD at ‘Baseline’ and ‘12 months’ stratified by AMD. Results are presented as mean ± SD. Two-way analysis of co-variance (ANCOVA) after adjustments by intervention (diet and physical activity). AMD × T interaction between adherence to the Mediterranean Diet and time. *t*-test for unpaired data in the differential values. Data points are significant when *p* < 0.05.

Figure 3 shows IFC levels at baseline and after 12 months of a nutritional intervention. The group that manages to adhere better to this diet achieved a significant decrease in the fat content in their liver, whereas no statistical differences were found in the group with lower adherence. The results of VO_2_ max in CST were represented in Figure 4. The obtained data reported an improvement in both groups after 12 months, although only significantly in the group with high AMD.

The results of polyphenols and MDA levels, and CAT and SOD enzymatic activities are shown in Table 2. MDA levels were significantly lower in both groups after 12 months. CAT activity was statistically significantly lower in patients with a in high ADM than in patients with a low AMD after 12 months of intervention. No significant differences were reported in polyphenols levels and in SOD activity between both groups.

The results of the ELISA and multiplex assays are shown in Table 3. MCP-1, CK-18, and leptin plasma levels were significantly lower and RvD1 levels were higher after 12 months in the group where the subjects acquired higher AMD. The levels of MPO, zonulin, and omentin were significantly lower after 12 months in both groups, regardless of the degree of AMD. No significant changes were observed in the levels of XOD, IL-1β, IL-1ra, IL-6, TNFα and chemerin.

**Figure 5:**
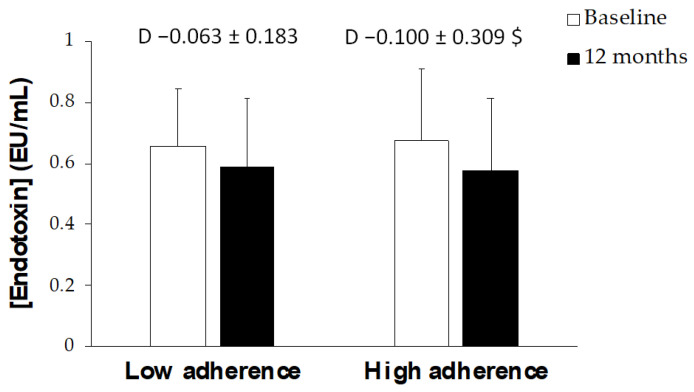
The concentration of endotoxin in patients with NAFLD at ‘Baseline’ and ‘12 months’ stratified by AMD. Results are shown as mean ± SD. Two-way analysis of co-variance (ANCOVA) after adjustments by intervention (diet and physical activity). AMD × T interaction between adherence to the Mediterranean Diet and time. *t*-test for unpaired data in the differential values. ^$^ Difference in means according to the differential (12-month values minus baseline values). Data points are significant when *p* < 0.05.

Figure 5 shows endotoxin concentration at baseline and after 12 months of intervention. The group which achieved a higher adherence to the Mediterranean diet presented a greater reduction in endotoxin levels than the group with a lower adherence improvement.

Table 4 shows crude and adjusted OR for the association between plasma biomarkers and AMD. Low adherence to the Mediterranean diet was established as the reference. OR crude and adjusted-1 analysis showed that higher AMD was considered a protective factor in front of CK-18 and EDA levels. AMD seemed to be a protective factor for IFC but after adjustment for intervention group, significance was lost. 

## 4. Discussion 

The most outstanding results of this study are the improvement of anthropometric, biochemical, oxidative, and inflammatory parameters in patients with NAFLD after 12 months of nutritional intervention. Although the patients followed different interventions, the results at 12 months were similar in the three groups. For this reason, and to delve into the possible causes responsible for this improvement, the patients have been grouped by the degree of improvement in adherence to the MedDiet between the start of the study and the intervention. The changes have been more evident in those patients with a greater improvement in the degree of ADM after the nutritional intervention than those with a lesser improvement in adherence. After 12 months of promoting a lifestyle intervention it has been shown, in both groups, an improvement in their physical fitness with respect to baseline since their VO_2_ max evaluated by Chester Step Test has increased. The same changes have been shown in systolic blood pressure. However, only participants with a higher ADM achieved significant weight loss and reduction in BMI. These results are in accordance with previous studies that showed how an energy-restricted MedDiet and exercise have effects on weight loss [26]. In addition, the results reported that the participants with a greater ADM improvement showed a significant decrease in IFC with respect the other group. The reduction in the liver fat contents has been reported to be related to a significant decrease in *de novo* lipogenesis and an increase in β-oxidation, without the need for a direct relation with weight or BMI loss [27]. Furthermore, some studies showed a direct relationship between physical training and a reduction of IFC, since exercise can regulate hepatic lipid metabolism, favoring their mobilization [28,29]. Regarding the lipid profile, triglycerides and total cholesterol have been decreased in both groups, but this reduction was more evident in subjects with a high AMD than subjects with low AMD. The present results are in line with other studies in which improved lipid profiles were also observed in subjects with high ADM with respect to subjects following a diet pattern more closely related to Western dietary patterns [30]. On the other hand, AST and ALT levels showed a decrease after one year of intervention, while GGT levels did not show changes. Similar results were previously obtained in NAFLD patients after 16 weeks of undergoing a MedDiet [31]. Another study also showed that the serum levels of liver enzymes, AST, ALT, and GGT, decreased after three and six months of intervention with MedDiet [32]. Moreover, there was a significant decrease in the current levels of CRP in the group with a higher AMD after 12 months of intervention when compared with the group with lower improvement in the ADM. Similar results were previously found, where a healthy dietary pattern such as the MedDiet was related to a significant reduction in CRP suggesting an attenuation of the inflammatory state [33].

Concerning oxidative stress markers, the present results revealed that plasma MDA levels, as a marker of lipid peroxidation, have been reduced in both groups after 12 months of intervention, especially in the group in which the participants had a high ADM and increased their physical capacity. These outcomes are in accordance with previous results showing that the concentrations of MDA were lower in the liver and serum of early NAFLD patients compared to advanced NAFLD [34]. The decrease in the MDA levels after the intervention is relevant since high levels of MDA are related to an increased expression of proinflammatory cytokines, interact with proteins and other lipids, and predispose to fibrosis of the liver parenchyma [35]. When analyzing the current variation of the antioxidant enzymes, CAT and SOD, no significant changes associated with the nutritional intervention were observed. Previous studies suggested that in the initial stages of the disease, an adaptive response of antioxidant defenses was produced, which; however, may be exhausted over time, and may be responsible for the absence of changes observed in the enzymes analyzed [35,36]. In addition, this absence of changes could also derive from the increase in other non-enzymatic antioxidants or from a reduction in the prooxidative state, with a decrease in the production of ROS [37]. Although an increased ADM pattern is related to higher ingestion of phenolic compounds, in the present study, after 1-year of intervention, the plasma levels of phenolic compounds did not show significant changes in any group. These results could be due to the fact that the maximum plasma levels of polyphenols are found after 1–2 h after ingestion, which could minimize the differences between groups after overnight fasting but also to their general poor bioavailability [38]. 

Current inflammatory markers such as XOD, IL-1β, IL-1ra, IL-6, TNFα, and chemerin levels did not report any changes between groups and time. After 12 months of following a dietary pattern based on the MedDiet and the promotion of physical activity, whereas MPO, omentin, and zonulin levels were reduced in the same way in the two groups. High MPO levels are associated with an increment of oxidative stress and inflammation which are characteristic of overweight and obesity [39]. Our results are in accordance with others studies showing that a better AMD and an increase in physical activity ameliorate the inflammatory profile and, therefore, decrease the levels of MPO [40,41]. Omentin is an anti-inflammatory adipokine mainly expressed and secreted from visceral adipose tissue [42]. A meta-analysis has reported the existence of heterogeneous results among the studies analyzed, although serum omentin level appears to be significantly lower in overweight subjects but not obese ones [43]. In addition, high levels of this adipokine were reported in patients with biopsy-proven NAFLD and showed a direct relationship with the degree of hepatocyte ballooning [44]. In this sense, the decrease observed in current groups may be related to a reduction in the BMI of the participants and in the systemic proinflammatory state associated with excessive fat accumulation. Zonulin is a described marker of intestinal permeability that has been reported to be increased in obesity and in NAFLD with a steep rise in NASH patients diagnosed by liver biopsy [45,46]. Since the liver receives most of its blood supply from the gut via the portal vein, it is one of the organs most exposed to gut-derived toxic factors [47]. In this regard, NAFLD has been reported to be associated with higher intestinal permeability and more disrupted tight junctions [48]. Thus, the observed decrease in plasma levels of zonulin may indicate a better intestinal state in parallel to a decrease in hepatic steatosis.

The main differential results between current groups were observed in the levels of MCP-1, CK-18, and leptin that decreased only in participants with a higher ADM after 1-year intervention, whereas levels of RvD1 increased. The overexpression of MCP-1 was related to an obese adipose tissue and high levels of this chemokine contribute to the macrophage infiltration into this tissue. Elevated levels of MCP-1 have been observed in patients with NAFLD and continue to increase in NASH [49]. In agreement with other studies, a high AMD is related to an improvement in the circulating levels of some chemokines including MCP-1 leading to a better inflammatory state [13,50]. CK-18 is a hepatic intermediate filament protein associated with the apoptosis of hepatocytes and it was studied as a non-invasive biomarker in NAFLD [51]. To current findings, CK-18 levels had a good correlation with steatosis grade, and both levels were directly related to specific enzymes activities [52,53]. Moreover, the present findings agree with a previous study that showed how a weight loss induced by a diet resulted in a significant decrease in CK-18 levels and a reduction in liver steatosis [54]. Leptin is a hormone secreted by adipose tissue which is involved in appetite regulation and the control of body weight [55]. Although there was controversy, recent studies revealed that increased levels were associated with the severity of hepatic steatosis [56,57]. The current results also evidenced a decrease in leptin levels in the group that achieved a greater reduction in intrahepatic content. On the other hand, RvD1 is an anti-inflammatory and pro-resolving mediator which has a positive protective effect on liver disease. In this sense, RvD1 levels increased according to the reduction in the degree of steatosis. In fact, it was seen how RvD1 had effects of hypoglycaemic and lipid-lowering in cell culture being an effective drug for NAFLD [58]. EDA is a hepatokine belonging to the superfamily of the TNF superfamily that has been reported to alter systemic insulin sensitivity in obesity and promote the activation of proinflammatory pathways, but also could aggravate hepatic steatosis by striking a balance between lipid deposition and elimination [59,60]. Although our results did not reveal significant changes in their levels, it can be appreciated a trend to reduced levels after a MedDiet and lifestyle intervention. These changes are in accordance with previous studies reporting that an increase in EDA levels was directly linked with inflammatory and steatosis states, even though their levels were not related to insulin resistance in human obesity [59,61].

Several studies reported that endotoxemia may be involved in the pathogenesis of NAFLD [62,63]. People with NAFLD showed an increased intestinal permeability, a prolonged orofecal transit time, and, a greater incidence of bacterial overgrowth in the small intestine [62,63]. In these patients, an increase in nonvirulent endotoxin-producing bacteria has been observed and may contribute to the pathology mediated by pro-inflammatory endotoxin-toll like receptor 4 (TLR4) crosstalk [64]. The current results revealed that the patients with a greater improvement in the degree of ADM after 12 months of intervention decreased the levels of endotoxin more with respect to those with a lower AMD. The results obtained in the current study agree on previous studies suggesting that modifying lifestyle could impact on reducing circulating levels of endotoxin [65,66]. Moreover, it had been observed that an increase in dietary fiber intake is inversely related to steatosis degree and bacterial endotoxin levels, suggesting that dietary fiber intake could be a potential target in NAFLD management [67].

Finally, the current findings from odd ratios showed how subjects who achieved a high AMD after 1 year of lifestyle intervention are protected in front of increased plasma levels of CK-18 and EDA and the percentage of IFC. 

## 5. Strengths and Limitations of the Study

The main strength of the current study is how an increase in AMD can reduce levels of oxidative stress and pro-inflammatory biomarkers after 1 year of intervention in patients with NAFLD. The main limitation of this study is that the sample size was relatively small, even though it was enough to evidence the existence of differences in the biomarker levels after a change of habits between subjects that achieve a high AMD, and those with a low AMD. Also, the analysis by gender could be interesting; however, the limited number of participants may make interpretation of the statistical results difficult.

## 6. Conclusions

The current study showed that a one-year nutritional intervention improved ain NAFLD features such as body mass index, IFC, liver enzymes, and pro-oxidant and pro-inflammatory status. There was also a decrease in the concentration of plasmatic endotoxin, suggesting an improvement in intestinal permeability. These health benefits were more evident in participants that improved their AMD to a greater extent.

## Figures and Tables

**Table 1:** Characteristics of participants ‘Baseline’ and ‘12 months’ stratified by AMD.

		Low Adherence (*n* = 32)	High Adherence (*n* = 35)	*p*-Value	*p*-Value
		Mean ± SD	Mean ± SD	AMD × T	Δ
Weight (kg)	Baseline	95.9 ± 13.8	93.5 ± 14.2	0.016	0.044
	12 months	93.2 ± 14.2	89.0 ± 13.1		
	Δ	−2.7 ± 4.7	−4.5 ± 5.7 ^$^		
BMI (kg/m^2^)	Baseline	33.6 ± 3.7	33.4 ± 4.2	0.336	0.049
	12 months	32.7 ± 4.1	31.9 ± 3.6		
	Δ	−1.0 ± 1.6	−1.6 ± 2.1 ^$^		
Systolic BP (mmHg)	Baseline	137 ± 17.0	138 ± 17.2	0.390	0.835
	12 months	130 ± 11.7	131 ± 10.6		
	Δ	−7.2 ± 16.2	−7.4 ± 14.6		
Diastolic BP (mmHg)	Baseline	81.8 ± 6.7	82.6 ± 10.7	0.958	0.738
	12 months	78.8 ± 8.3	81.7 ± 6.7		
	Δ	−3.0 ± 9.2	−0.98 ± 8.2		
Glucose (mg/dL)	Baseline	107 ± 20.8	116 ± 34.6	0.372	0.224
	12 months	107 ± 26.3	107 ± 25.7		
	Δ	−0.80 ± 16.5	−8.60 ± 21.5		
HbA1c (%)	Baseline	5.8 ± 0.6	6.1 ± 1.0	0.024	0.636
	12 months	5.7 ± 0.7	6.0 ± 0.9		
	Δ	−0.1 ± 0.5	−0.1 ± 0.5		
Triglycerides (mg/dL)	Baseline	173 ± 62.8	213 ± 111	0.041	0.022
	12 months	163 ± 71.3	186 ± 109		
	Δ	−10.0 ± 65.1	−27.6 ± 136.0 ^$^		
HDL-cholesterol (mg/dL)	Baseline	41.1 ± 8.8	40.9 ± 7.4	0.851	0.349
	12 months	42.7 ± 10.9	42.6 ± 9.2		
	Δ	1.6 ± 7.4	1.7 ± 5.8		
LDL-cholesterol (mg/dL)	Baseline	131 ± 29.3	125 ± 31.9	0.450	0.812
	12 months	120 ± 30.3	121 ± 37.2		
	Δ	−10.6 ± 30.8	−4.37 ± 25.0		
Cholesterol total (mg/dL)	Baseline	209 ± 36.8	216 ± 60.9	0.810	0.031
	12 months	199 ± 45.2	202 ± 46.7		
	Δ	−10.4 ± 35	−14.9 ± 72.6 ^$^		
AST (IU/L)	Baseline	24.4 ± 7.1	26.7 ± 8.6	0.124	0.024
	12 months	22.0 ± 6.3	23.1 ± 6.5		
	Δ	−2.5 ± 5.1	−3.6 ± 9.0 ^$^		
ALT (IU/L)	Baseline	34.9 ± 19.8	40.6 ± 26.4	0.027	0.796
	12 months	27.7 ± 10.3	28.2 ± 14.6		
	Δ	−7.20 ± 18.7	−12.3 ± 21.8		
GGT (IU/L)	Baseline	42.5 ± 23.0	39.4 ± 16.6	0.698	0.235
	12 months	37.7 ± 21.7	36.6 ± 23.8		
	Δ	−4.87 ± 15.7	−2.84 ± 22.6		
CRP (mg/dL)	Baseline	0.5 ± 0.5	0.5 ± 0.6	0.230	0.006
	12 months	0.4 ± 0.4	0.3 ± 0.2		
	Δ	−0.1 ± 0.3	−0.2 ± 0.5 ^$^		

Abbreviations: BMI: Body mass index, systolic BP: systolic blood pressure; diastolic BP: diastolic blood pressure, HbA1c: Glycated haemoglobin A1c, HDL-cholesterol: high-density lipoprotein, LDL-cholesterol: low-density lipoprotein, AST: aspartate aminotransferase, ALT: alanine aminotransferase, GGT: gamma glutamyl transferase, CRP: c-reactive protein, IFC: intrahepatic fat content, SD: Standard deviation. Results are expressed as mean ± SD. Two-way analysis of co-variance (ANCOVA) was performed after adjustments by the intervention (diet and physical activity) or a Kruskal–Wallis test according to the case. *t*-test for unpaired data was performed on the differential data (12-month values minus reference values) or with U Mann–Whitney test according to the case. ^$^ Difference in means according to the differential (12-month values minus baseline values). Data points in bold are significant, *p* < 0.05.

**Table 2:** Oxidative stress markers in the plasma of participants ‘Baseline’ and ‘12 months’ stratified by AMD.

		Low Adherence (n = 32)	High Adherence (n = 35)	*p*-Value	*p*-Value
		Mean ± SD	Mean ± SD	AMD × T	Δ
Antioxidants					
Phenolic compounds (mM)	Baseline	0.3 ± 0.1	0.3 ± 0.2	0.829	0.074
	12 months	0.3 ± 0.2	0.3 ± 0.2		
	Δ	0.0 ± 0.2	0.1 ± 0.2		
Oxidative damage					
MDA (nM)	Baseline	2.0 ± 1.0	1.8 ± 0.7	0.037	0.050
	12 months	1.0 ± 0.4 *	0.8 ± 0.3 *		
	Δ	−1.0 ± 1.0	−1.1 ± 0.7 ^$^		
Enzyme activities					
CAT (k(s^−1^)/L blood)	Baseline	54.5 ± 17.4	47.5 ± 13.5	0.040	0.545
	12 months	58.7 ± 22.1	46.4 ± 23.3 ^#^		
	Δ	4.2 ± 28.3	−1.1 ± 28.0		
SOD (pkat/L blood)	Baseline	288 ± 61.0	276 ± 81.7	0.383	0.733
	12 months	254 ± 73.7	245 ± 94.6		
	Δ	−34.1 ± 112.0	−25.1 ± 200.0		

Abbreviations: MDA: malondialdehyde; CAT: catalase; SOD: superoxide dismutase. SD: Standard deviation. Results are expressed as mean ± SD. Two-way analysis of co-variance (ANCOVA) was performed after adjustments by intervention (diet and physical activity) or a Kruskal–Wallis test according to the case. * Difference in means between participants in time (baseline and 12 months). ^#^ Difference in means between groups (low adherence and high adherence). *t*-test for unpaired data was performed on the differential data (12-month values minus reference values) or with U Mann–Whitney test according to the case. ^$^ Difference in means according to the differential (12-month values minus baseline values). Data points in bold are significant, *p* < 0.05.

**Table 3:** Inflammatory markers of participants ‘Baseline’ and ‘12 months’ stratified by AMD.

		Low Adherence (n = 32)	High Adherence (n = 35)	*p*-Value	*p*-Value
		Mean ± SD	Mean ± SD	AMD × T	Δ
XOD (ng/mL)	Baseline	0.4 ± 0.2	0.4 ± 0.1	0.455	0.391
	12 months	0.3 ± 0.2	0.4 ± 0.2		
	Δ	−0.1 ± 0.2	0.0 ± 0.2		
IL-1β (pg/mL)	Baseline	1.3 ± 0.6	1.2 ± 0.5	0.340	0.704
	12 months	1.3 ± 0.5	1.2 ± 0.4		
	Δ	0.0 ± 0.3	0.0 ± 0.2		
IL-1ra (pg/mL)	Baseline	130 ± 307	131 ± 126	0.090	0.054
	12 months	85.7 ± 85.9	90.1 ± 108		
	Δ	−44.5 ± 279	−41.3 ± 135		
IL-6 (pg/mL)	Baseline	4.2 ± 0.4	4.2 ± 0.3	0.239	0.578
	12 months	4.2 ± 0.4	4.2 ± 0.3		
	Δ	0.0 ± 0.4	−0.0 ± 0.2		
TNFα (pg/mL)	Baseline	3.9 ± 0.7	3.9 ± 0.5	0.159	0.113
	12 months	3.9 ± 0.5	3.9 ± 0.5		
	Δ	0.0 ± 0.4	−0.0 ± 0.5		
MCP-1 (pg/mL)	Baseline	13.4 ± 12.6	10.7 ± 10.3	0.021	0.105
	12 months	13.3 ± 10.3	8.5 ± 5.8 *		
	Δ	0.1 ± 11.6	−2.2 ± 9.8		
MPO (ng/mL)	Baseline	4.0 ± 2.5	4.6 ± 2.8	0.045	0.343
	12 months	2.8 ± 1.5*	2.9 ± 2.04 *		
	Δ	−1.0 ± 2.5	−1.5 ± 3.6		
RvD1 (pg/mL)	Baseline	143 ± 70	138 ± 37	0.063	0.018
	12 months	167 ± 144	166 ± 120 *		
	Δ	24.5 ± 165	27.8 ± 124 ^$^		
CK-18 (pM)	Baseline	91.6 ± 68.4	112 ± 76	0.002	0.448
	12 months	83.3 ± 52.7	79.5 ± 55.9 *		
	Δ	−6.64 ± 64.3	−26.0 ± 57.2		
Zonulin (ng/mL)	Baseline	5.0 ± 2.4	5.8 ± 3.6 ^#^	0.033	0.048
	12 months	1.9 ± 1.2 *	2.57 ± 1.6 *		
	Δ	−3.1 ± 2.1	−3.5 ± 4.2 ^$^		
EDA (pg/mL)	Baseline	507 ± 184	618 ± 246 ^#^	0.015	0.162
	12 months	460 ± 149	468 ± 202		
	Δ	−47.3 ± 186	−150 ± 201		
Omentin (ng/mL)	Baseline	156 ± 49.3	149 ± 57.6	<0.001	0.226
	12 months	85.2 ± 34.4 *	80.5 ± 37.9 *		
	Δ	−69.1 ± 52.8	−67.8 ± 48.7		
Chemerin (ng/mL)	Baseline	86.4 ± 17.1	85.6 ± 13.9	0.222	0.856
	12 months	90.4 ± 25.0	84.4 ± 13.8		
	Δ	4.0 ± 19.6	−1.2 ± 17.0		
Leptin (ng/mL)	Baseline	18.2 ± 7.9	19.2 ± 6.62	0.036	0.049
	12 months	15.1 ± 5.2	15.1 ± 6.4 *		
	Δ	−3.0 ± 7.8	−3.7 ± 5.4 ^$^		

Abbreviations: XOD: xanthine oxidase; IL-1β: interleukin-1β; IL-1ra: interleukin-1ra; IL-6: interleukin-6; TNFα: tumour necrosis factor α; MCP-1: monocyte chemoattractant protein 1; MPO: myeloperoxidase; RvD1: resolving D1; CK-18: Cytokeratin-18; EDA: ectodysplasin-A. SD: Standard deviation. Results are expressed as mean ± SD. Two-way analysis of co-variance (ANCOVA) was performed after adjustments by intervention (diet and physical activity) or a Kruskal–Wallis test according to the case. * Difference in means between participants in time (baseline and 12 months). ^#^ Difference in means between groups (low adherence and high adherence). *t*-test for unpaired data was performed on the differential data (12-month values minus reference values) or with U Mann-Whitney test according to the case. ^$^ Difference in means according to the differential (12-month values minus baseline values). Data points in bold are significant, *p* < 0.05.

**Table 4:** Association between plasma biomarkers and ADM in participants after a 12-Month of lifestyle intervention.

		Low Adherence (n = 32)	High Adherence (n = 35)	
		OR (95% CI)	OR (95% CI)	*p*-Value
XOD	Crude OR	1.00 (ref.)	1.393 (0.640–3.032)	0.404
	OR Adjusted 1	1.00 (ref.)	1.438 (0.622–3.328)	0.396
IL-1β	Crude OR	1.00 (ref.)	0.543 (0.263–1.119)	0.098
	OR Adjusted 1	1.00 (ref.)	0.679 (0.317–1.453)	0.318
IL-1ra	Crude OR	1.00 (ref.)	0.474 (0.220–1.020)	0.056
	OR Adjusted 1	1.00 (ref.)	0.501 (0.227–1.107)	0.087
IL-6	Crude OR	1.00 (ref.)	0.907 (0.445–1.847)	0.788
	OR Adjusted 1	1.00 (ref.)	0.845 (0.404–1.769)	0.655
TNFα	Crude OR	1.00 (ref.)	0.860 (0.415–1.780)	0.683
	OR Adjusted 1	1.00 (ref.)	0.983 (0.456–2.118)	0.965
MCP-1	Crude OR	1.00 (ref.)	0.782 (0.384–1.593)	0.498
	OR Adjusted 1	1.00 (ref.)	1.090 (0.498–2.386)	0.830
MPO	Crude OR	1.00 (ref.)	0.611 (0.290–1.288)	0.195
	OR Adjusted 1	1.00 (ref.)	0.602 (0.278–1.301)	0.197
RvD1	Crude OR	1.00 (ref.)	1.371 (0.680–2.766)	0.377
	OR Adjusted 1	1.00 (ref.)	1.245 (0.597–2.597)	0.559
CK-18	Crude OR	1.00 (ref.)	0.326 (0.157–0.676)	0.003
	OR Adjusted 1	1.00 (ref.)	0.330 (0.152–0.714)	0.005
Zonulin	Crude OR	1.00 (ref.)	1.432 (0.687–2.983)	0.338
	OR Adjusted 1	1.00 (ref.)	1.341 (0.629–2.858)	0.447
EDA	Crude OR	1.00 (ref.)	0.206 (0.097–0.437)	<0.001
	OR Adjusted 1	1.00 (ref.)	0.239 (0.111–0.517)	<0.001
Omentin	Crude OR	1.00 (ref.)	1.000 (0.486–2.059)	1.000
	OR Adjusted 1	1.00 (ref.)	1.014 (0.473–2.173)	0.971
Chemerin	Crude OR	1.00 (ref.)	0.600 (0.293–1.227)	0.162
	OR Adjusted 1	1.00 (ref.)	0.597 (0.284–1.253)	0.173
Leptin	Crude OR	1.00 (ref.)	1.062 (0.521–2.166)	0.868
	OR Adjusted 1	1.00 (ref.)	1.123 (0.537–2.350)	0.759
Phenolic compounds	Crude OR	1.00 (ref.)	1.432 (0.687–2.983)	0.338
	OR Adjusted 1	1.00 (ref.)	1.595 (0.745–3.412)	0.229
MDA	Crude OR	1.00 (ref.)	0.755 (0.351–1.622)	0.471
	OR Adjusted 1	1.00 (ref.)	0.751 (0.338–1.669)	0.482
CAT	Crude OR	1.00 (ref.)	0.611 (0.290–1.288)	0.195
	OR Adjusted 1	1.00 (ref.)	0.629 (0.289–1.366)	0.241
SOD	Crude OR	1.00 (ref.)	1.418 (0.672–2.992)	0.359
	OR Adjusted 1	1.00 (ref.)	1.552 (0.709–3.397)	0.271
IFC	Crude OR	1.00 (ref.)	0.456 (0.229–0.910)	0.026
	OR Adjusted 1	1.00 (ref.)	0.516 (0.252–1.056)	0.070
Endotoxin	Crude OR	1.00 (ref.)	0.818 (0.340–1.970)	0.654
	OR Adjusted 1	1.00 (ref.)	0.830 (0.344–2.005)	0.679

Abbreviations: OR: odds ratio; OR adjusted 1: odds ratio after adjustments by intervention (diet and physical activity); ref.: reference; XOD: xanthine oxidase; IL-1β: interleukin-1β; IL-1ra: interleukin-1ra; IL-6: interleukin-6; TNFα: tumor necrosis factor α; MCP-1: monocyte chemoattractant protein 1; MPO: myeloperoxidase; RvD1: resolving D1; CK-18: cytokeratin-18; EDA: ectodysplasin-A.

## Data Availability

There are restrictions on the availability of data for this trial due to the signed consent agreements around data sharing, which only allow access to external researchers for studies following the project’s purposes. Requestors wishing to access the trial data used in this study can make a request to pep.tur@uib.es.
